# Comparative multi-omics systems analysis reveal the glycolysis / gluconeogenesis signal pathway play an important role in virulence attenuation in fish-derived GBS YM001

**DOI:** 10.1371/journal.pone.0221634

**Published:** 2019-08-26

**Authors:** Yu Liu, Liping Li, Ting Huang, Rui Wang, Wanwen Liang, Qiong Yang, Aiying Lei, Ming Chen

**Affiliations:** Guangxi Academy of Fishery Sciences, Nanning,China,P.R. China; National Cheng Kung University, TAIWAN

## Abstract

*Streptococcus agalactiae*(GBS) is a seriously threat to the farmed tilapia, and oral vaccination was considered to be the most desirable means which requires deep understanding of virulence mechanism of the fish-derived GBS. Our previous genome study of the fish-derived attenuated strain YM001 showed that there were two large deletions in YM001 compared to its parental virulent strain HN016. In this study, a combined transcriptomic and proteomic analysis was performed on YM001 and HN016 strains, and the important genes were verified by RT-qPCR in bacteria strains and infected-tilapia tissues. Overall, we have shown that a total of 958 genes and 331 proteins were significantly differential expressed between YM001 and HN016. By functional annotation of these DEGs and DEPs, genes that were enriched in pentose phosphate pathway(pgm, ptsG, pgi pfkA, fbaA and FBP3) and pyruvate metabolism pathway(pdhA, pdhB, pdhC and pdhD) were identifed as important candidate genes for leads low growth ability in attenuated strain, which may be an important reasons leading virulence attenuation in the end. The expression levels the candidate genes in pentose phosphate pathway and pyruvate metabolism pathway were significant differential expressed in tilapia’ brain and spleen when infected with YM001 and HN016. Our study indicated that the pentose phosphate pathway and pyruvate metabolism pathway that affecting the growth of the strain may be one of the important reasons for the virulence attenuation in HN016.

## Introduction

*Streptococcus agalactiae*(GBS) is a gram-positive bacterium, which caused streptococcal disease result in large-scale infections and death of many seawater and freshwater fishes[1–4]. Tilapia is an important economically farmed fish worldwide which was particularly vulnerable to the disease[5,6]. Since 2009, large-scale streptococcal outbreaks occurred continuously in China with high mortality(30–90%), and more than 90% of the clinical strains are GBS[7–9]. Feeding infected fish with antibiotic-medicated feed is a general practice in control of the streptococcosis[10]. However, the use of the aquaculture antibiotics is not standard in applications caused adverse effects, such as environmental pollution, drug residue and resistant strains[11]. Therefore, alternative control methods, such as vaccination, are urgently needed to control streptococcosis.

Vaccination is more ideal approach which had advantages in safety and convenience which had becoming the best means to control streptococcosis at present[12,13]. In the major vaccination routes, oral vaccination was considered to be the most desirable route[14]. Compared with the traditional inactivated whole bacterium and subunit GBS vaccines, oral vaccines have many advantages such as convenient vaccination, less stressful to fish, and broad inoculation range[15–20]. At present, oral attenuated vaccine has become a hot spot in oral vaccine development, for it can greatly avoid intestinal enzymatic degradation and reach the organs including spleen, kidney, etc., resulting in sustained immune responses[21]. However, current developed oral GBS vaccine had some defects such as weak immune response, short protection period and narrow protection range due to the lack of suitable attenuated vaccine strains[22,23]. We had previously developed an attenuated GBS vaccine for tilapia via continuous passage in vitro, which can provided good immune protection by oral immunization. The attenuated strain YM001(Ia,ST7) showed good safty, stability and highly immunogenic[24]. However, due to the diversity of tilapia GBS strains and differences in distribution[25], the protection range of YM001 is restricted, which requires more candidate strains. And It’s not feasible in practice and application to use our previous metheds to obtain strains due to the long period of preparation. Therefore, deep study of virulence mechanism of GBS is required to rapid obtaining attenuated vaccine strains. Although the GBS strains of human and fish sources contain similar virulence factors [26,27], some studies had shown that there are differences in the virulence mechanisms between human and fish GBS[26,28]. Therefore, it is necessary to systematically study the virulence mechanisms of fish-derived GBS.

Previous genome study of the fish-derived attenuated GBS strain YM001 (Ia,ST7) showed that there were two large deletions(D1, 5,832 bp; D2, 11,116 bp) in YM001 compared to its parental virulent strain HN016 (Ia,ST7)[27]. Virulence test showed that YM001 failed to cause disease or death to tilapia at the dose of 10^9^ CFU/fish when HN016 was lethal to tilapia at the dose of 10^3^ CFU/fish[24]. In this study, a combined transcriptome and proteome analysis was performed on YM001 and HN016. Aim to further explosed the virulence mechanism of fish-derived GBS and provided a theoretical basis for the rapid preparation of attenuated vaccine strains.

## Materials and methods

### Bacterial strains and fishes

*S*.*agalactiae* strain HN016 was isolated from a moribund cultured tilapia with typical clinical and pathogenic characteristics of meningitis (Hainan,China, 2010), belonged to GBS serotype Ia, multilocus sequence type seven (ST7). The HN016 strain was used as starting material to generate an attenuated strain by continuous passage in vitro, and the 840th passage was named strain YM001(Ia, ST7)[24].

Non-infected Nile tilapia(*Oreochromis niloticus*) with average weight of 400±15.10g were provided by the National Tilapia Seed Farm(Nanning, Guangxi, China), which were confirmed to be negative for bacterial infection by bacteriological analysis of the brain and kidney samples. The fishes were monitored twiced a day with a formulated diet (Tongwei Feed Company, Nanning, China).

### Ethics approval and consent to participate

Animal experiments were conducted in strict accordance with the Chinese animal experiment ethical inspection, under project licence number: GXU2015039, approved by the Guangxi University, CHINA.

### RNA-sequencing

The strains were cultured followed our previous methods[24]. Briefly, the strain HN016 and YM001 were removed from -80°C refrigerator, then streaked onto a 5% sheep blood agar plate, and cultured at 30°C for 24 h. A single colony was then picked up, inoculated into 10 mL of TSB medium, and cultivated at 30°C by shaking. After 12 h, 1.0 mL of bacteria was inoculated into fresh 10 mL of TSB medium and cultured continuously by shaking for another 12 h. Collected the bacterium solution and stored in RNAprotect®bacteria Reagent(QIAGEN,Germany), then sent to the Novogene Co, LTD (Beijing) for RNA-seq and proteome analysis.

All following Kits listed were used according manufacture’s recommendations. Total RNA was extracted by using Tiangen RNA prep Pure Plant Kit (Tiangen Biomart, Beijing). RNA degradation and contamination was monitored on 1% agarose gels. RNA purity was checked using the NanoPhotometer® spectrophotometer (IMPLEN, CA,USA). RNA concentration was measured using Qubit® RNA Assay Kit in Qubit® 2.0 Flurometer (Life Technologies, CA, USA). RNA integrity was assessed using the RNA Nano 6000 Assay Kit of the Agilent Bioanalyzer 2100 system (Agilent Technologies, CA, USA). A total amount of 3 μg RNA per sample was used as input material for the RNA sample preparations. Sequencing libraries were generated using NEBNext® Ultra^™^ Directional RNA Library Prep Kit for Illumina® (NEB, USA) following manufacturer’s recommendations[29] and index codes were added to attribute sequences to each sample. Quantified cDNA libraries (effective concentration, 2 nM) were sequenced using an Illumina HiSeq platform. Clean reads were obtained by removing low-quality reads, and reads containing poly-N and adapters were mapped back onto the reference genome sequence (NCBI, Accession NO.NZ_CP011325.1) by using STAR (v2.5.1b). HTSeq v0.6.0 was used to count the reads numbers mapped to each gene[30]. Transcript abundance was measured as a unit of the expected number of fragments per kilobase of transcript per million mapped reads (FPKM)[31]. Differential expression analysis of two conditions/groups (two biological replicates per condition) was performed using the DESeq2 R package (1.10.1)[32]. DESeq2 provide statistical routines for determining differential expression in digital gene expression data using a model based on the negative binomial distribution. The resulting P-values were adjusted using the Benjamini and Hochberg’s approach for controlling the false discovery rate. Genes with an adjusted P-value <0.05 found by DESeq2 were assigned as differentially expressed. The statistical enrichment of DEGs in Gene Ontology was performed using GOseq[33], DEGs in KEGG pathways was performed using KOBAS v2.0[34]. Raw data had been submitted to National Center for Biotechnology Information(NCBI) with the accession numbers <SRR7841403> and <SRR7841410> for YM001 and HN016, respectively.

### Protein extraction and peptide preparation

The samples were individually milled to a power in a mortar with liquid nitrogen. We then mixed 150 mg of the powder from each sample with 1 ml of lysis buffer containing Tris-base (pH 8), 8M Urea, 1% SDS, complete protease inhibitor cocktail (Sigma) in a glass homogenizer. The homogenate was incubated on ice for 20 min and then centrifuged at 12000 g for 15 min at 4°C. The supernatant was transferred to a clean tube, and protein concentration was determined with a Bradford assay. And then added 4 volumes 10 mM DTT in cold acetone to a sample extract, vortexed well, placed samples at -20°C for 2 h or overnight. Centrifuged and collected pellet to wash twice with cold acetone. Finally dissolved the pellet by dissolution buffer containing Tris-base (pH = 8), 8M Urea.

### iTRAQ labeling, HPLC fractionation and LC-MS/MS analysis

Desalted peptides were labeled with iTRAQ reagents (iTRAQ® Reagent-8PLEX Multiplex Kit,Sigma), following the manufacturer’s instructions (AB Sciex, Foster City, CA). For 0.1 mg of peptides, 1 unit of labeling reagent was used. Peptides were dissolved in 20 μl of 0.5 M triethylammonium bicarbonate solution (TEAB, pH 8.5), and the labeling reagent was added to 70 μl of isopropanol. After incubation for 1 h, the reaction was stopped with 50 mM Tris/HCl (pH 7.5). Differently labeled peptides were mixed equally and then desalted in 100 mg SCX columns (strata-x-c, Phenomenex: 8B-S029-EBJ).

A 600 microgram iTRAQ-labeled peptide mix was fractionated using a C18 column (waters BEHC18 4.6 × 250 mm, 5 μm) on a Rigol L3000 HPLC operating at 1ml/min. The column oven was set as 50°C. Mobile phases A (2% acetonitrile, 20mM NH4FA, adjusted pH to 10.0 using NH3·H2O) and B (98% acetonitrile, 20mM NH4FA, adjusted pH to 10.0 using NH3·H2O) were used to develop a gradient elution. The solvent gradient was set as follows: 3–8% B, 5min; 8–18% B, 12 min; 18–32% B, 11 min; 32–45% B, 7 min; 45–80% B, 3 min; 80% B, 5 min; 80–5%, 0.1min, 5% B, 7 min. The tryptic peptides were monitored at UV 214 nm. Eluent was collected every minute and then merged to 15 fractions. The samples were dried under vacuum and reconstituted in 20μl of 0.1% (v/v) FA, 3% (v/v) acetonitrile in water for subsequent analyses.

Fractions from the first dimension RPLC were dissolved with loading buffer and then separated by a C18 column (150 μm inner-diameter, 360 μm outer-diameter × 15 cm, 1.9μm C18, Reprosil-AQ Pur, Dr. Maisch). Mobile phase A consisted of 0.1% formic acid in water solution, and mobile phase B consisted of 0.1% formic acid in acetonitrile solution; a series of adjusted 60 min gradients according to the hydrophobicity of fractions eluted in 1D LC with a flow rate of 300 nL/min was applied. Q-Exactive HF-X mass spectrometer was operated in positive polarity mode with capillary temperature of 320°C. Full MS scan resolution was set to 60000 with AGC target value of 3e6 for a scan range of 350–1500 m/z. A data-dependent top 40 method was operated during witch HCD spectra was obtained at 15000 MS2 resolution with AGC target of 1e5 and maximum IT of 45 ms, 1.6 m/z isolation window, and NCE of 30, dynamically excluded of 60s.

### The identification and quantitation of protein

The resulting spectra from each fraction were searched separately against the “Run2_Streptococcus_agalactiae_GCF_001190805.1_ASM119080v1_protein.fasta” database by Proteome Discoverer2.2 software(Thermo Fisher Scientific). The searched parameters as follows: A mass tolerance of 10 ppm for precursor ion scans and a mass tolerance of 0.02 Da for the product ion scans were used. Carbamidomethyl was specified in PD 2.2 as fixed modifications. Oxidation of methionine, acetylation of the N-terminus and iTRAQ 8-plex of tyrosine, lysine were specified in PD 2.2 as variable modifications. A maximum of 2 miscleavage sites were allowed.

For protein identification, protein with at least 1 unique peptide was identified at FDR less than 1.0% on peptide and protein level, respectively. Proteins containing similar peptides and could not be distinguished based on MS/MS analysis were grouped separately as protein groups. Reporter Quantification (iTRAQ 8-plex) was used for iTRAQ quantification. The protein quantitation results were statistically analyzed by Mann-Whitney Test, the significant ratios, defined as p < 0.05 and |log2FC| >*(ratio > * or ratio < * [fold change, FC]), were used to screen the differentially expressed proteins (DEP).

The mass spectrometry proteomics data have been deposited to the ProteomeXchange Consortium via the MASSIVE with the dataset identifier < PXD011206>.

### Data analysis of proteome

Gene Ontology (GO) and InterPro (IPR) analysis were conducted using the interproscan-5 program against the non-redundant protein database (including Pfam, PRINTS, ProDom, SMART, ProSiteProfiles, PANTHER) [35], and the databases COG (Clusters of Orthologous Groups) and KEGG (Kyoto Encyclopedia of Genes and Genomes) were used to analyze the protein family and pathway. The probable interacting partners were predicted using the STRING-db server (http://string.embl.de/) based on the related species. STRING is a database of both known and predicted protein-protein interactions[36]. The enrichment pipeline [37] was used to perform the enrichment analysis of GO, IPR, COG and KEGG, respectively.

### Histopathological analysis

The experiment was refers to our previous methods[38]. At 48 h post-infection, the brain, liver, spleen, head kidney, and intestine of infected tilapia were collected for tissue injury pathological analysis. Samples were collected from the freshly dead fish. For live fish group, 25 fishes were anesthetize with high concentration of benzocaine for 5 min. When the fishes had no response to touch and light, sacrificed for sampling. The whole process takes no more than 10 minutes. Following standard fixation in 10% neutral buffered formalin and sample processing in paraffin wax blocks, paraffin sections (6μm thick) were stained with hematoxylin and eosin (H&E) for light microscopy observations.

### Validation of gene expression level by real-time quantitative PCR (RT-qPCR)

The RT-qPCR was used to verify the expression levels of 16 candidate genes between YM001 and HN016 in culture strains and different infected tilapia tissues. The recA[39] gene was selected as a standardization control, and the specific primers used to amplify the candidate genes were designed using Primer 5 software. Briefly, total RNA was extracted respective from HN016,YM001 strains and infected tilapia tissues(brain, liver, spleen, head kidney, and intestine, respectively). then reverse transcribed into cDNA by HiScript® II 1st Strand cDNA Synthesis Kit (+gDNA wiper) (Vazyme, Nanjing). Real-time qPCR was performed in a DNA Engine Chromo 4 real-time system (BioRad) with HiScript II One Step qRT-PCR SYBR Green Kit (Vazyme, Nanjing). The expression of genes was calculated as relative expression to recA using the 2(-Δ Δ C(T)) method and samples were analyzed in triplicates.

## Results

### Overviews of RNA transcriptomic and quantitative proteomic analyses profiles

**Fig 1:**
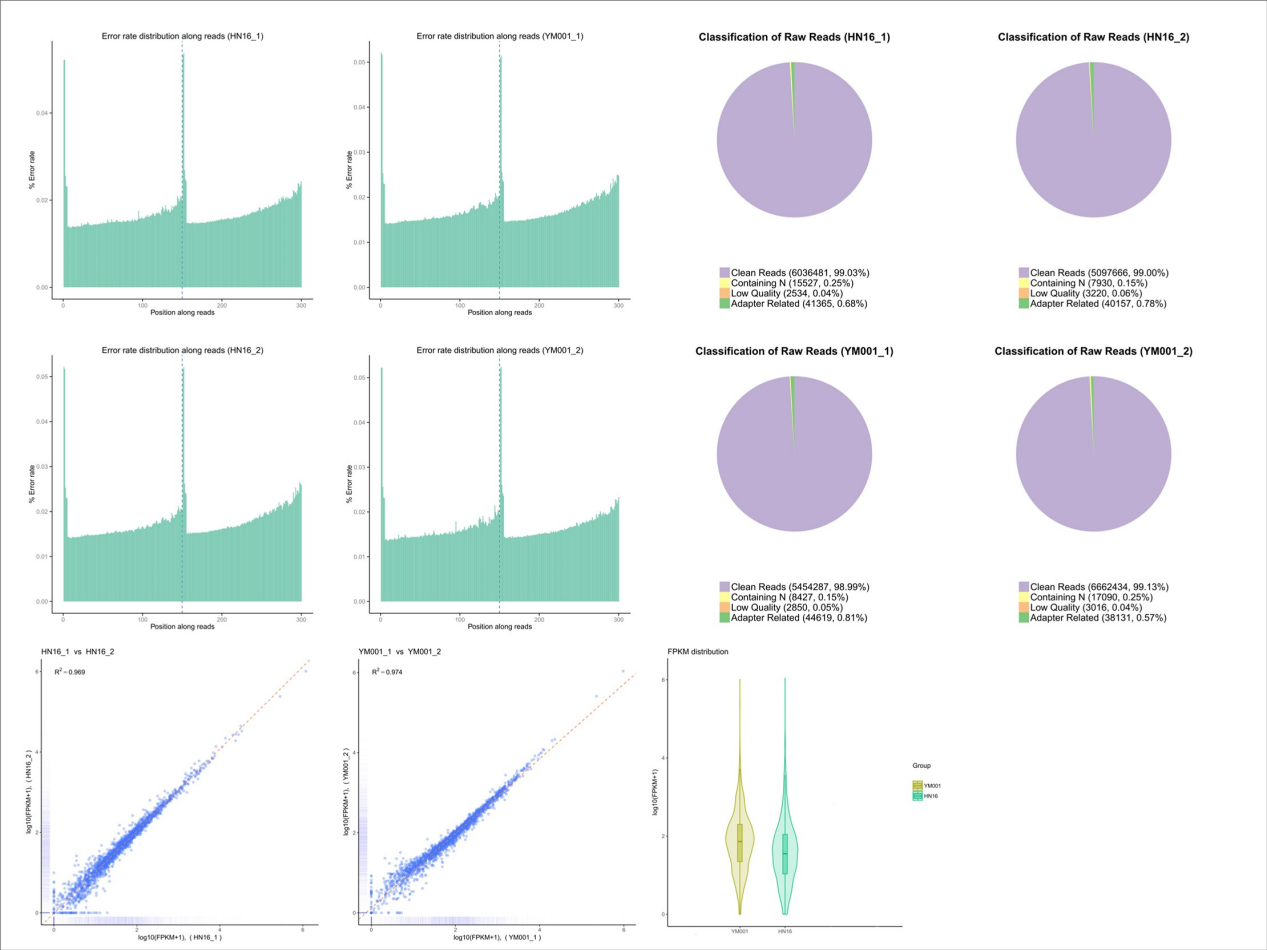
Quality control of the multi-omics analysis.

**Fig 2:**
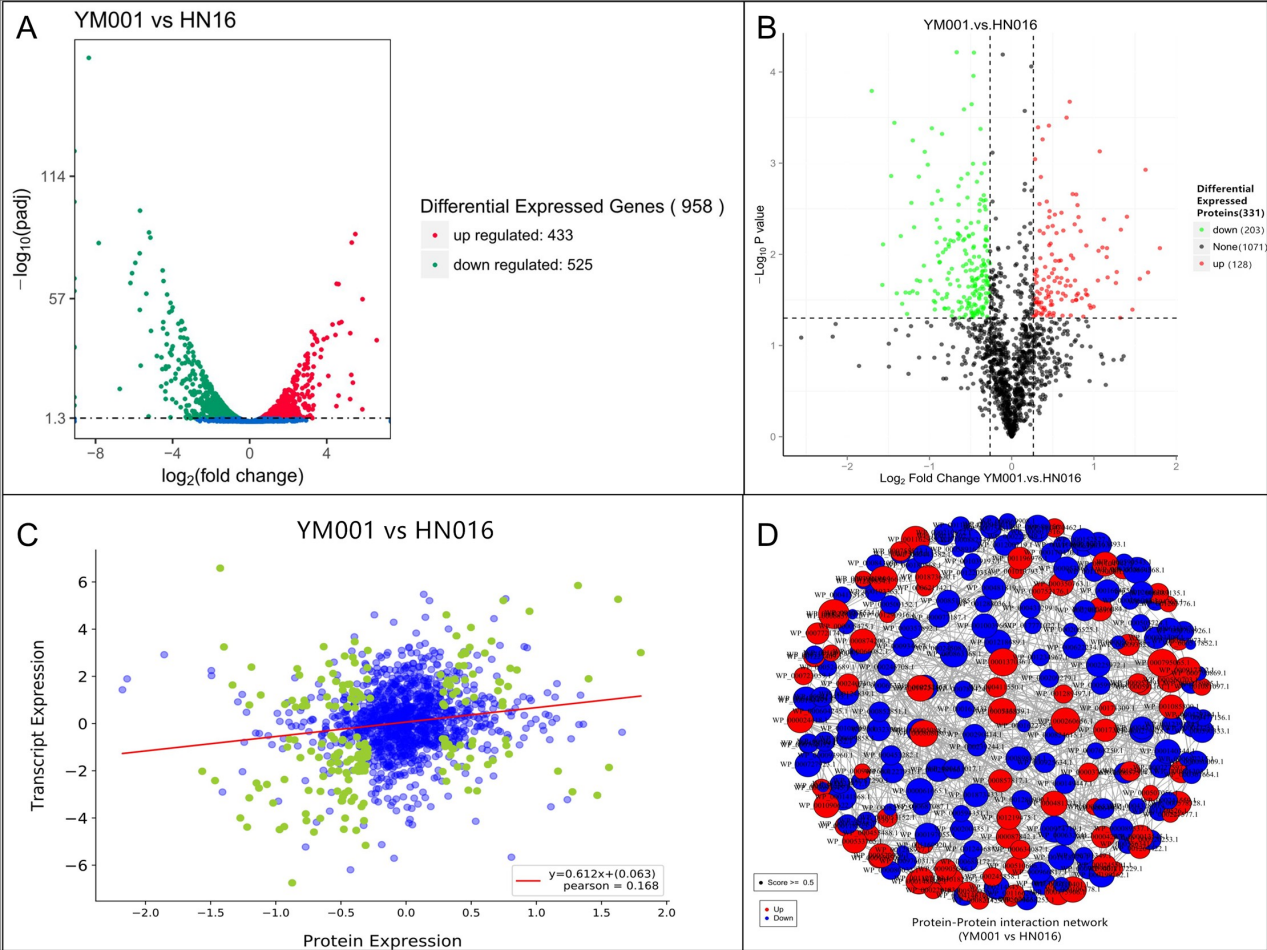
Differential expression analysis of transcriptome and proteome. A shown the differential expressed genes between YM001 and HN016. B shown the differential expressed proteins between YM001 and HN016. C shown the correlation analysis of the transcriptome and proteome. D shown the interaction between the DEPs. Error bars represent the standard deviation. *indicates that the difference in gene expression between YM001 and HN016 reached the significant level of 0.01≤P≤ 0.05. ** indicates that the difference in gene expression between YM001 and HN016 reached the significant level of P≤ 0.01.

In RNA-seq transcriptomic analyses, a total of 46,951,468 raw reads (11,020,366 and 13,441,342 for YM001 as well as 12,191,814 and 10,297,946 for HN016, respectively) were generated, and 46,501,736 clean reads were obtained after cleaning and quality checks. Approximately 97.77% (95.45–99.59%) of the mapped reads were acquired from the RNA-seq experiment of which 96.95% (94.61–98.83%) were mapped to unique genomic locations(Fig 1, S1 Table). A total of 958 genes (433 up-regulated and 525 down-regulated)(Fig 2A) and 331 proteins(128 up-regulated and 203 down-regulated)(Fig 2B) were significantly altered (P<0.05) in YM001 vs HN016 group. Fig 2D showed the interaction work between the DEPs.

### Funtional analysis of DEGs and DEPs

**Fig 3:**
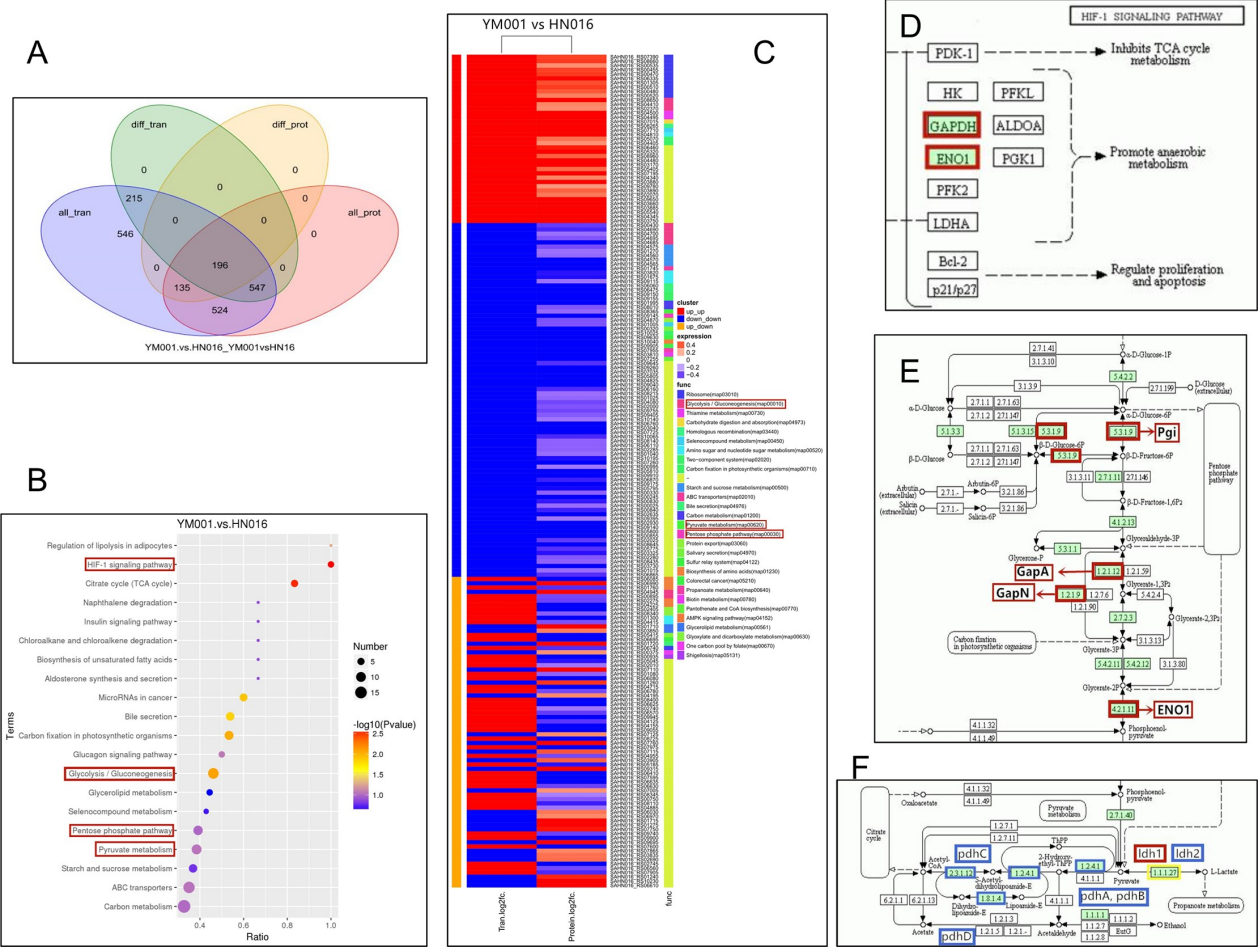
Funtional analysis of DEGs and DEPs, and RT-qPCR validation. A shown the genes (proteins) with similar alteration trends at both mRNA and protein levels. B shown the most enriched signaling pathways. C shown the 196 significant DEGs (DEPs) annotated into KEGG pathways. D shown the HIF-1 signaling pathway. E shown the pentose phosphate pathway. F shown the pyruvate metabolism pathway.

Comparison between the transcriptomic and proteomic analyses identified 196 proteins (Fig 3A) with similar alteration trends at both mRNA and protein levels. GO and KEGG pathway were performed on the DEGs and DEPs. The 196 significant DEGs/DEPs were enriched in different signaling pathways (Fig 3C), such as HIF-1 signaling pathway, glycolysis / gluconeogenesis, pentose phosphate pathway and pyruvate metabolism.etc.(Fig 3B).

Combined transcriptome and proteome analyses shown that HIF-1 signaling pathway(GAPDH and ENO1; Fig 3D), pentose phosphate pathway(pgm, ptsG, pgi pfkA, fbaA and FBP3; Fig 3E) and pyruvate metabolism pathway(pdhA, pdhB, pdhC and pdhD; Fig 3F) were the main differences between the two strains.

### RT-qPCR validation

**Fig 4:**
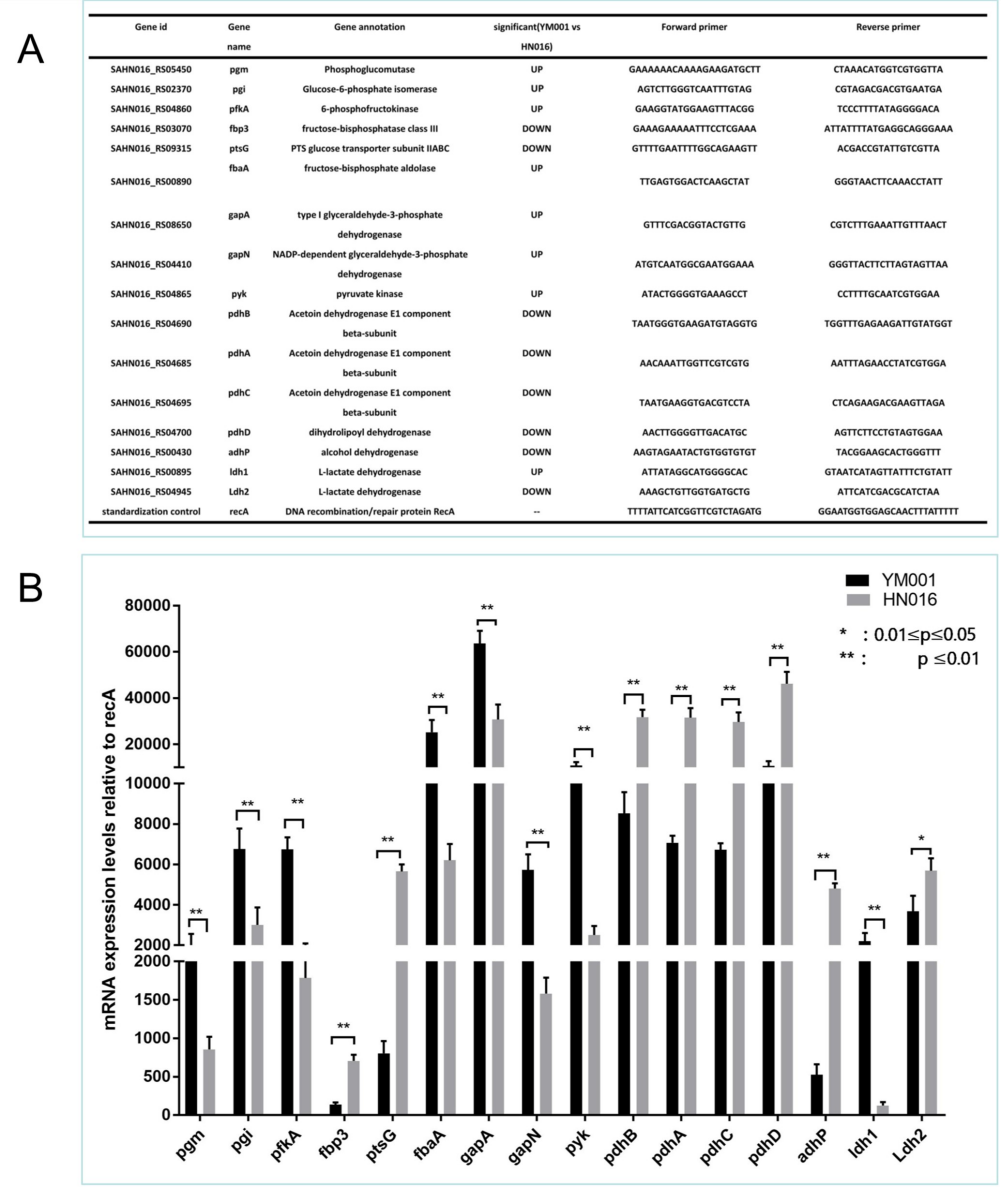
RT-qPCR validation of DEGs and DEPs. A shown the informations of the 16 candidated genes. B shown the result of the RT-qPCR validation of the candidated genes.

**Fig 5:**
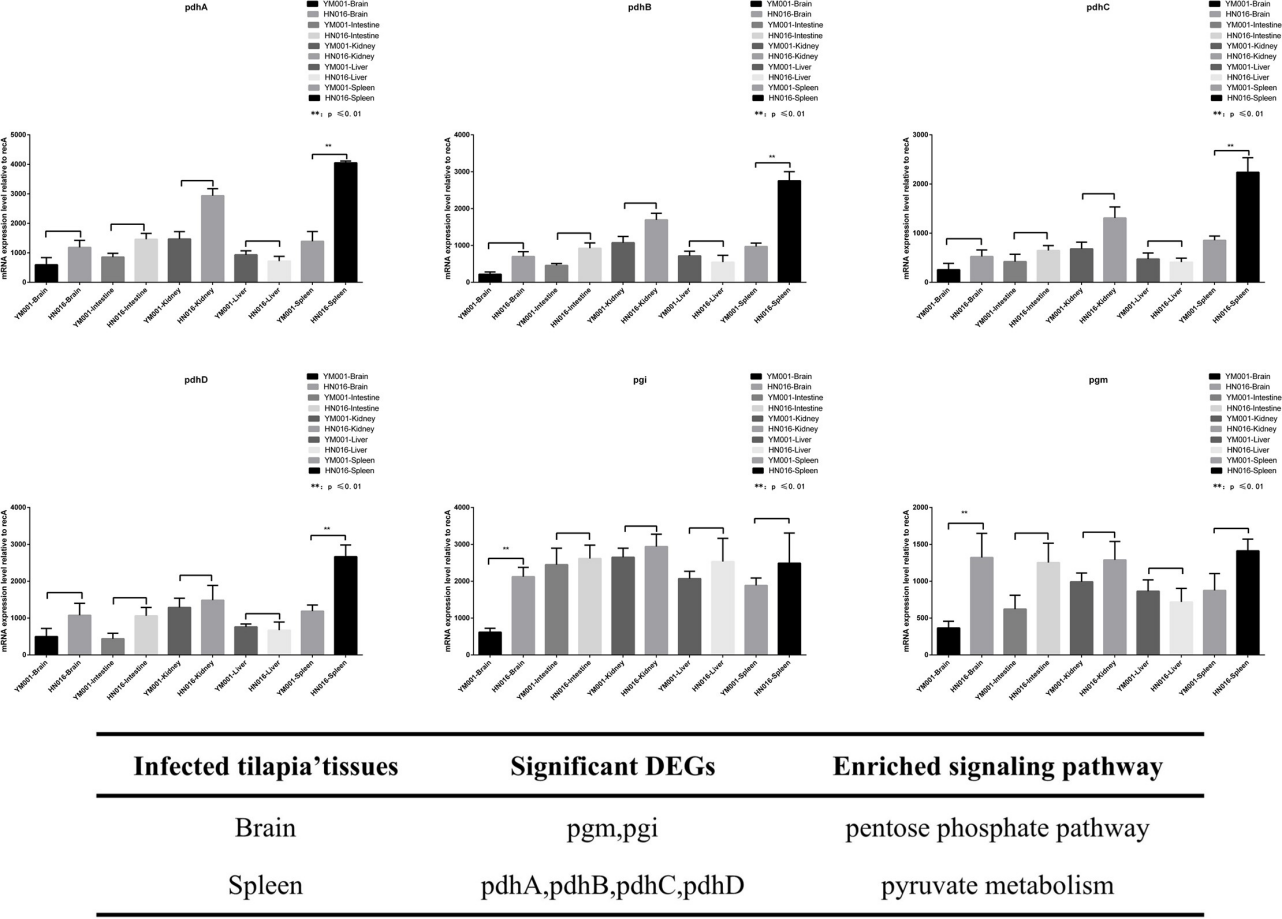
RT-qPCR validation. Figure shows the expressed levels of the candidated genes in HIF-1 signaling pathway, pentose phosphate pathway and pyruvate metabolism pathway in different tissue of the tilapia when infected with YM001 and HN016, respectively. Error bars represent the standard deviation. *indicates that the difference in gene expression between YM001 and HN016 reached the significant level of 0.01≤P≤ 0.05. ** indicates that the difference in gene expression between YM001 and HN016 reached the significant level of P≤ 0.01.

The expression level of the 16 significant DEGs in HIF-1 signaling pathway, pentose phosphate pathway and pyruvate metabolism pathway were identified by RT-qPCR. The results was consistent with transcriptome analysis(Fig 4). The candidated genes above were significant different expressed in five different tissues(brain, liver, spleen, kidney and intestine) of the tilapia which infected with YM001 and HN016,respectively. Pgm and pgi gene were significant differential expressed in brain while pdhA, pdhB, pdhC and pdhD were significant differential expressed in spleen between two infection groups(Fig 5).

### Histopathological analysis

**Fig 6:**
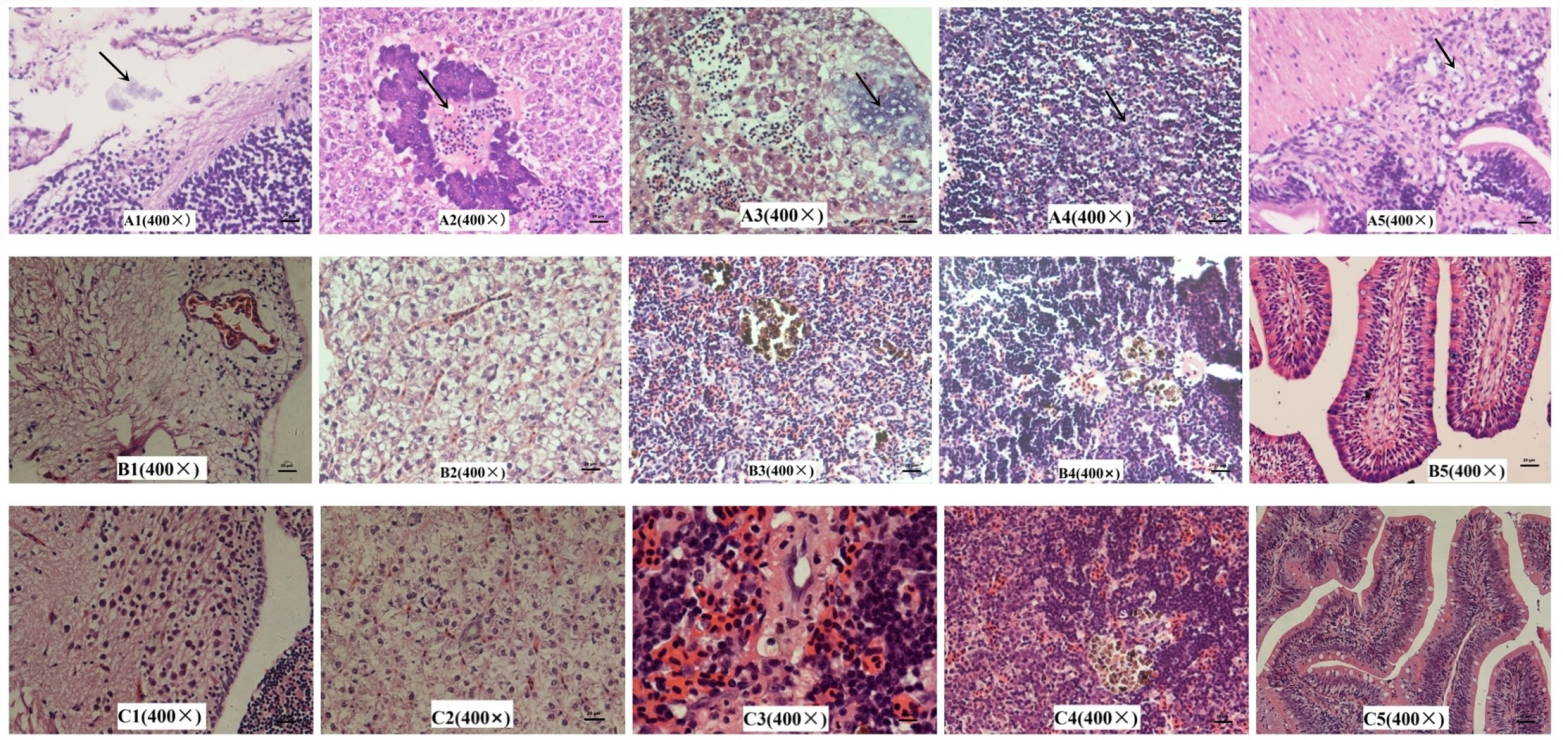
Histologic sections. A、B、C showed the histopathological results of brain, liver, spleen, head kidney, and gut from tilapia injected with HN016, YM001 and control group, respectively. The black arrow points out the places gathered with blue dye-stained Streptococcus particles.

Histopathological examination showed that there were severe lesions in the examined tissues of tilapia infected by HN016(Fig 6). For the brain tissue, the changes included edema, loose and thickening in meninges, disperse from brain matrix, interstitial inflammatory cell infiltration, capillary congestion, bleeding, large number of visible blue dye-stained Streptococcus particles. In the liver tissue, the lesions included congestion of central vein and hepatic sinus, vascular wall damage, endothelial cell necrosis and shedding, the inflammatory cell infiltration around central vein and pancreas, as well as the stained Streptococcus particles in the pancreas and liver sinus. Other changes including liver cells degeneration, necrosis,and disintegration were also observed. The changes from spleen included serious disorder of tissue structure, red blood cell infiltration in white pulp area, disappearance of lymphoid tissue structure, lymphocytes necrosis and number reduction, a large number of blue-stained Streptococcus granules in necrotic area, and scattered hemosiderin deposition. In the head kidney tissue, it was observed a lot of visible blue dye-stained Streptococcus particles.Intestinal serosal boundary was blurred, and visible blue dye-stained Streptococcus granules were observed in the serosa, myometrium and submucosa. In contrast, no obvious histopathological changes were observed in fish injected with YM001 and control group. The histopathological results demonstrated the different virulence characteristics of HN016 and YM001.

## Discussion

The virulence mechanism of fish-derived GBS is still remains unknown, thus systemic analysis is in need. In this study, integrative bioinformatic analysis of both transcriptomic and proteomic data of both virulent strain and attenuated strain revealed some key signaling pathways with many key genes which might be an important reason of the virulence attenuation in YM001.

GBS is a facultative anaerobe which colonized in many tissues including the gastrointestinal and genitourinary tracts, brain, blood, liver, kidney, mammary gland, etc[40], and transmitted by blood[41]. The ability of bacterial pathogens to adapt to often-changing environments within a single host is critical to their growth and survival. Studies have shown that oxygen affects the infectivity and virulence of GBS [42]. Bacteria respond to harmful oxidative stress by producing antioxidant molecules, such as NADPH[43]. Pentose phosphate pathway(PPP) is complementary and alternative to glycolysis. PPP is the main producer of NADPH and pentose phosphates. NADPH regenerates reduced glutathione and reduced thioredoxin that protect hemoglobin and essential thiol groups from oxidation. Pentose phosphates are utilized for nucleoside, DNA, and RNA synthesis.[44]. We have shown that pgm, ptsG, pgi pfkA, fbaA and FBP3 genes are significant differential expressed in YM001 and HN016. The enzymes encoded by these DEGs catalyze one of the reactions in the PPP, which leads PPP was significant up-regulated in YM001 than HN016. Meantime, the GAPDH and the downstream enzymes enolase ENO1 were up-regulated in YM001 than HN016, which involves in the HIF-1 signaling pathway and promote the anaerobic metabolism[45,46]. The NADPH and pentose phosphates produced by the PPP can effectively enhance the growth and survival of GBS, and eliminate the harm of the high concentration of reactive oxygen species(ROS) produced by bacteria during rapid proliferation period to host cells[47]. It may be an important reasons that YM001 can survived and induce immune protection in tilapia.

Pyruvate is located at a metabolic junction of assimilatory and dissimilatory pathways and represents a switch point between respiratory and fermentative metabolism. The pyruvate dehydrogenase complex (PDHC) is considered the primary routes of pyruvate conversion to acetyl-CoA for aerobic respiration[48]. PDHC deficiency is one of the most common causes of mitochondrial dysfunction[49]. We have shown that three of the main enzymes, pyruvate decarboxynase(pdhA/pdhB), dihydrolipoamide transacetylace(pdhC) and dihydrolipoamide dehydrogenase(pdhD) were significant down-regulated in YM001. The mitochondrial dysfunction caused by PDHC deficiency may be the main reason that YM001 grows more slowly than HN016 when culture in vitro. Therefore, the decrease of growth ability leads to the virulence attenuation in YM001.

Histologic sections showed that the tilapia had obvious pathological changes infected with HN016, while no obvious change when infected with YM001. We had identified the expression level of the 16 significant DEGs mentioned above in five tissues(brain, intestine, kidney, liver and spleen) of tilapia which were infected YM001 and HN016, respectively. 10 genes were significant differential expressed in YM001 and HN016 with vary widely among different tissues. Among them, pgm and pgi genes were significant down-regulated in YM001, which was contrary to the results of multi-omics. Study had shown that GPI was identified as novel adhesive moonlighting proteins[50]. In order to produce meningitis, GBS must interact with and breech the blood-brain barrier (BBB), which consists of specialized human brain microvascular endothelial cells (hBMECs). [51]. Therefore, strains with high expression of GPI have stronger adhesion ability, resulting in stronger virulence. PDHC was significant down-regulated in YM001 in spleen, consistent with multi-omics analysis. Spleen is a multifunctional organ of fish, which plays an important role in the hematopoiesis and immunity. The low proliferative ability of YM001 in spleen leads low virulence to tilapia and induces host immune protection. It’s suggests that the physiological state and activated functions of GBS may be different in different tissues of the infected tilapia at the same time.

Integrative bioinformatic analysis of both transcriptomic and proteomic data of GBS attenuated strain YM001 and its virulental strain HN016 reveals that pentose phosphate pathway (pgm, ptsG, pgi pfkA, fbaA and FBP3) and pyruvate metabolism pathway(pdhA, pdhB, pdhC and pdhD) were the main differences between the two strains. The differences in PPP and PDHC leads low growth ability in attenuated strain, which may be an important reasons leading virulence attenuation in the end. This study provided a comprehensive analysis of the fish-derived GBS strains which were virulent and non-virulent to tilapia, respectively. Furthermore, some potential targets for antibacterial drug discovery were identified.

## Supporting information

S1 Table The summary of the sequence data and Gene expression quantifications (FPKM). (DOC)
