# Supplementary material for: Comparative multi-omics systems analysis reveal the glycolysis / gluconeogenesis signal pathway play an important role in virulence attenuation in fish-derived GBS YM001
Source: PLoS One. 2019 Aug 26;14(8):e0221634. doi: 10.1371/journal.pone.0221634 (PMC6709914; doi:10.1371/journal.pone.0221634)
Supplement: S1 Table — (DOC) [file pone.0221634.s001.doc]

| Sample name | YM001-1 | YM001-2 | HN016-1 | HN016-2 |
| --- | --- | --- | --- | --- |
| Raw reads | 11020366 | 13441342 | 12191814 | 10297946 |
| Clean reads | 10908574 | 13324868 | 12072962 | 10195332 |
| Clean bases | 1.64 G | 2 G | 1.81 G | 1.53 G |
| Error rate(%) | 0.02 | 0.02 | 0.02 | 0.02 |
| Q20(%) | 97.92 | 98.11 | 97.94 | 97.79 |
| Q30(%) | 93.49 | 93.92 | 93.5 | 93.18 |
| GC content(%) | 38.01 | 37.7 | 37.06 | 37.37 |
| FPKM Interval（0~1） | 195（9.02%） | 175（8.09%） | 237（10.96%） | 260（12.02%） |
| FPKM Interval（1~3） | 73（3.37%） | 60（2.77%） | 126（5.83%） | 137（6.33%） |
| FPKM Interval（3~15） | 315（14.56%） | 293（13.55%） | 455（21.04%） | 397（18.35%） |
| FPKM Interval（15~60） | 468（21.64%） | 555（25.66%） | 618（28.57%） | 606（28.02%） |
| FPKM Interval（＞60） | 1112（51.41%） | 1080（49.93%） | 727（33.61%） | 763（35.28%） |

**Table S1. The summary of the sequence data and Gene Expression Quantifications(FPKM)**
